# Development and validation of the Thai Halitosis Associated Life-Quality Test (T-HALT): an evaluation of psychometric properties

**DOI:** 10.1186/s12903-024-04926-y

**Published:** 2024-10-08

**Authors:** Yodhathai Satravaha, Katkarn Thitiwatpalakarn, Supakit Peanchitlertkajorn, Supatchai Boonpratham, Chaiyapol Chaweewannakorn, Kawin Sipiyaruk

**Affiliations:** Department of Orthodontics, Faculty of Dentistry, Mahidol University, 6 Yothi Road, Ratchathewi, Bangkok, 10400 Thailand

**Keywords:** Halitosis, Quality of life, Oral health, Psychometrics

## Abstract

**Background:**

Halitosis appears to have significant impacts on quality of life, necessitating reliable assessment tools. The Halitosis Associated Life-Quality Test (HALT) has been validated in various populations, but not among Thai people. While HALT provides a valuable foundation, there is a need for a culturally adapted and expanded instrument for the Thai context. Consequently, this study aimed to develop and validate a comprehensive questionnaire for assessing halitosis-related quality of life in Thai populations, incorporating a Thai version of HALT (T-HALT) as a core component.

**Materials and methods:**

This cross-sectional study involved 200 dental patients at Mahidol University. The original HALT was translated into Thai using forward-backward translation. Cultural adaptation and psychometric properties of T-HALT were evaluated through multiple approaches. Content validity was ensured through expert reviews, while face validity was assessed by patient feedback. Reliability was examined via test-retest and internal consistency measures. Criterion and discriminant validity was evaluated by correlating T-HALT scores with self-perceived halitosis and volatile sulfur compound (VSC) measurements, respectively. VSCs were quantified using the OralChroma™ device, which analyzes breath samples collected directly from patients’ mouths. Construct validity was assessed through exploratory (EFA) and confirmatory factor analysis (CFA), providing insights into the questionnaire’s underlying structure.

**Results:**

T-HALT demonstrated excellent internal consistency (Cronbach’s alphas = 0.940–0.943) and test-retest reliability (ICC = 0.886). Criterion validity was supported by a significant correlation between T-HALT scores and self-perceived halitosis (*r* = 0.503, *P* < 0.001). Discriminant validity was confirmed by the absence of a significant correlation between T-HALT scores and VSC levels (*r* = 0.071, *P* = 0.32). EFA revealed a four-factor structure, which was subsequently confirmed by CFA. However, Items 1 and 7 were excluded due to poor standardized factor loadings.

**Conclusion:**

T-HALT demonstrates good reliability and validity for assessing halitosis-related quality of life in Thai populations. It performs well as a unidimensional measure, but its multidimensional application requires modifications. Future research should validate a modified version excluding Items 1 and 7 across diverse Thai populations, potentially enhancing its cultural specificity.

**Supplementary Information:**

The online version contains supplementary material available at 10.1186/s12903-024-04926-y.

## Introduction

Halitosis, often described as foul breath odor, significantly impacts individuals beyond its physical manifestations. Emerging from various oral and systemic issues, such as inadequate plaque control and gastrointestinal problems, halitosis is fueled by odoriferous components, mainly volatile sulfur compounds (VSCs) produced by oral bacteria within the oral cavity through the enzymatic reaction [1]. The repercussions of halitosis extend far beyond physical discomfort, profoundly affecting social interactions, self-esteem, mental well-being, relationships, work productivity, and sense of belonging [2]. Existing evidence consistently highlights the severe psychosocial consequences of halitosis, including heightened embarrassment, anxiety, social withdrawal, diminished self-esteem, and depression [3–5]. Such consequences can perpetuate a cycle of isolation and distress, highlighting the urgent need for prompt diagnosis and effective management of halitosis.

The identification of halitosis employs a range of examination techniques, encompassing both subjective and objective methods. Objective measurements are conducted in various ways, including gas chromatography, beta-galactosidase activity quantification, salivary incubation test, ammonia monitoring, and the cysteine challenge test [6]. Among these, gas chromatography is particularly favored for its high objectivity, precision, and sensitivity, enabling the discrete detection of minute compounds such as VSCs in the gas phase [7–9]. However, previous research indicates that in the absence of patient complaints, there is often a presumption of no need for diagnosing halitosis, even if objective measurements suggest elevated VSC levels [10]. This discrepancy highlights a critical aspect of halitosis assessment, as the impact on quality of life may be more significantly influenced by self-perceived halitosis than by objectively measured halitosis.

Various instruments are available for assessing the impact of halitosis on quality of life. The Medical Outcomes Short-Form Health Survey Questionnaire (SF-36) offers a broad evaluation of health-related quality of life following halitosis therapy [11]. Additionally, the Oral Health Impact Profile (OHIP) and the Oral Impacts on Daily Performance (OIDP) questionnaires, specifically designed to explore the multidimensional effects of oral disorders, facilitate the examination of halitosis’s correlation with oral health-related quality of life (OHRQoL) [3]. Despite their applicability for assessing various aspects of oral health, these instruments may lack the specificity required to fully capture the impact of halitosis on OHRQoL.

The Halitosis Associated Life-Quality Test (HALT), a condition-specific questionnaire designed to assess the relationship between oral malodor and oral health-related quality of life (OHRQoL), was developed to provide a standardized instrument [12]. This tool facilitates comparisons of the psychosocial impact of halitosis across diverse populations. Validation efforts for the HALT questionnaire have been performed with multiple populations, including Polish [13], Brazilian [14], and Romanian [15]. In the Asian context, validation has been limited to the Chinese population [16]. Notably, these validation studies have yielded varying results. The Brazilian version demonstrated unidimensionality [14], whereas the Romanian iteration demonstrated four distinct factors [15]. Moreover, the Chinese adaptation necessitated the exclusion of certain items [16]. These inconsistencies highlight the significance of further validations of HALT to refine its efficacy.

Studies in Thailand have investigated the association between oral diseases and quality of life [17–19]. While questionnaires like OHIP and SF-36 have been employed in these studies, there is a lack of research utilizing the HALT questionnaire. HALT is considered more specific and superior to other instruments in assessing halitosis-related quality of life due to its focused design and comprehensive coverage of halitosis impacts. Despite HALT holding significant potential for application [20, 21], its translation and validation in the Thai context have not been previously undertaken. Meaningful interpretation of questionnaire-derived data necessitates rigorous validation of the instrument against objective measures within the target population, ensuring both validity and reliability of the research findings [22]. Consequently, this study was conducted to develop and validate a comprehensive questionnaire for assessing halitosis-related quality of life in Thai populations, incorporating a Thai version of HALT (T-HALT) as a core component. Through a rigorous validation process, a robust tool for accurately capturing and understanding self-perceived halitosis within the population of interest was developed. Furthermore, this article provides a methodical outline of the comprehensive validation process employed, elucidating various techniques utilized to assess the questionnaire validity and reliability.

## Materials and methods

### Research design

This study employed a quantitative cross-sectional survey design to develop and validate a robust questionnaire specifically designed to explore self-perceived halitosis in the Thai population, with HALT serving as an integral section of this comprehensive instrument. The research was divided into two phases: Phase 1: Cultural Adaptation and Phase 2: Psychometric Validation. Following ethics approval, data collection for Phase 1 was conducted over a four-week period from June to July 2023. Subsequently, that of Phase 2 encompassed a six-month duration, from August 2023 to January 2024. To ensure a thorough validation process, various techniques to assess the validity and reliability of the questionnaire were employed and critically discussed.

### Research participants

The research population comprised dental patients aged 18 years or older attending the Dental Hospital, Faculty of Dentistry, Mahidol University. To enhance the generalizability of findings and comprehensively evaluate T-HALT, participants both with and without a history of halitosis were included. However, participants were excluded if they had consumed tea, coffee, juice, or chewing gum, or had smoked within one hour preceding the assessment. Those who had used mouth rinse or perfumes within the preceding 12 h were also excluded. Additionally, individuals were excluded from participation if they were not fluent in Thai, had literacy difficulties, visual or hearing impairments, memory problems, or intellectual disabilities. These exclusion criteria were also implemented in previous literature of HALT [14–16]. These criteria ensured a standardized assessment environment and participant capability, enhancing the reliability of the study results.

Participant recruitment was facilitated using convenience sampling [23]. This method involved selecting participants who were readily accessible rather than randomly chosen from a larger population, which could lead to sampling bias. Nonetheless, increasing the sample size can enhance the statistical power of the convenience sample and reduce the time and costs required for data collection. Phase 1 was conducted with 30 participants for cultural adaptation. A small sample size was generally adequate for estimating the Intraclass Correlation Coefficient (ICC) in test-retest reliability, as this reliability measure was typically evaluated in an initial study with a smaller cohort [24–26]. For Phase 2, the sample size was determined based on the factor analysis requirements, which suggest 10 to 20 individuals per questionnaire item [27, 28]. Consequently, a minimum sample size of 200 participants was designated for this phase.

### Data collection procedures

#### Gas chromatography

The OralChroma™ (CHM-2, Abimedical, Abilit Corp., Osaka, Japan), a portable gas chromatograph, was utilized to quantify VSCs from participants, offering an objective and precise measurement of halitosis [10]. The protocol for VSC collection employed a precise methodology. Initially, researchers introduced a syringe into the participant’s oral cavity, ensuring the plunger was completely compressed. Subjects were then directed to seal their lips, engage in nasal respiration, and refrain from oral movement for a duration of 60 s. Participants were instructed to avoid lingual contact with the syringe tip. The procedure continued with the operator retracting the plunger, followed by a recompression to facilitate air mixing within the oral cavity. A final plunger retraction was then performed to secure the breath specimen for analysis. Subsequently, the syringe tip was meticulously cleansed to remove any saliva that might contaminate the sample. The plunger was then precisely adjusted to collect 1 mL of the breath sample, ensuring standardization across all measurements. Finally, the collected gas sample was injected into the OralChroma™ device in a single, swift stroke for immediate analysis. This methodical process ensured the integrity of the sample from collection to analysis, minimizing potential sources of error and maximizing the reliability of the VSC measurements.

#### Self-administered questionnaire

The development of the questionnaire was informed by a comprehensive review of relevant literature in the field of halitosis-related quality of life [12, 14–16]. The items in the ‘halitosis-related quality of life’ section were adopted from the original HALT questionnaire. The HALT questionnaire comprised 20 items, each evaluated on a 6-point Likert scale. The scale ranged from 0 (No problem), 1 (Very mild problem), 2 (Mild or slight problem), 3 (Moderate problem), 4 (Severe problem), and 5 (Problem as bad as it can be). The total score was calculated by summing the ratings across all 20 items. Higher total scores indicated a more significant negative impact of halitosis on the individual’s quality of life [12]. Authorization to translate and use the HALT questionnaire in this study was obtained from the corresponding author of the original HALT article [12]. Originally developed in English, the questionnaire underwent a rigorous translation process into Thai to enhance participant comprehension and minimize potential response bias. The questionnaire consisted of three sections, with two sections undergoing item modifications during the validation process to improve psychometric properties. Both the English and Thai versions of the questionnaires are provided as supplementary materials (Supplementary 1 and Supplementary 2, respectively).

### Validation techniques and their analytic approaches

**Fig. 1:**
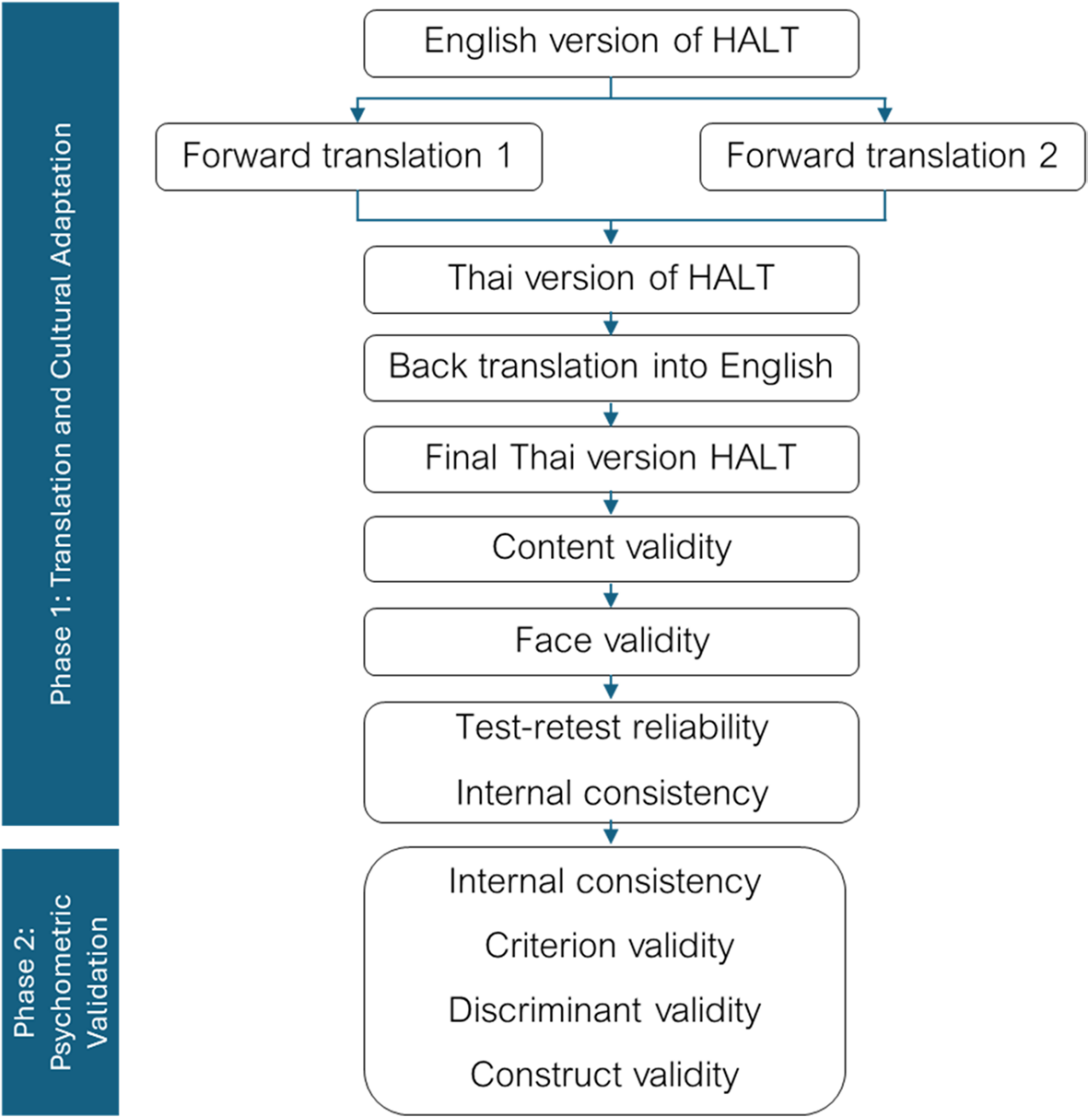
The process of translation and validation

To ensure the psychometric integrity of the questionnaire, a series of validation techniques were implemented, including translation validity, content validity, face validity, criterion validity, discriminant validity, and construct validity. Additionally, test-retest reliability and internal consistency were evaluated to establish the instrument’s reliability. The process of translation and validity techniques is illustrated in Fig. 1. The following sections discuss the implementation of these validation and reliability techniques, including their respective analytical approaches. All statistical analyses were performed using SPSS and AMOS (Version 29, IBM Corp.).

#### Translation validity

With HALT serving as an entire section of the questionnaire, the original version underwent a rigorous translation into Thai using a scientific procedure to ensure accuracy. Following translation validity guidelines [29, 30], forward and backward translation, and cross-cultural adaptation were employed. The process comprised several key steps. Initially, two researchers (YS and KT) proficient in both English and Thai independently translated the English version of HALT into Thai, resulting in two forward translations. Subsequently, the two translations were compared and merged through discussion, yielding a synthesized Thai version of HALT. This Thai version was back-translated into English by a bilingual professional translator without prior knowledge of the original HALT questionnaire. The back-translation was then compared to the original HALT questionnaire by the researchers (KT, YS, and KS), noting any discrepancies in synonymous words. Throughout this process, strict adherence was maintained to preserve the integrity of the instructional content, ensuring that no alterations, additions, or omissions were made. For further details, see Supplementary 3.

#### Content validity

To ensure content validity, three experts in the field of halitosis evaluated the content to ensure adequate representation of the domain of interest. The index of item-objective congruence (IOC) was employed as a quantitative measure to assess the degree of agreement between each item and the hypothesized underlying construct. Each expert rated each item as follows: a score of 1 if it clearly measured the objective, a score of -1 if it clearly did not measure the objective, or a score of 0 if it was unclear in measuring the objective. The IOC was calculated by summing up the content validity scores (ΣR) and dividing by the number of experts (N). Additionally, qualitative evaluations involved considering suggestions from experts during iterative revisions of the questionnaire. These revisions were conducted until each item achieved an IOC higher than 0.5, as recommended in previous literature. This value was used in previous literature [31–33]. For further details, see Supplementary 4.

#### Test-retest reliability

The test-retest reliability assessment in Phase 1 involved 30 participants completing the questionnaire and then again after one week to ensure consistency over time [24, 26]. Data collection procedures were methodically maintained to ensure consistency and standardization across both test and retest administrations. This process is grounded in the assumption that in the absence of changes in the patient status, responses from the test and retest questionnaires should be consistent [34]. The concordance of data obtained from the two questionnaires was evaluated using the intraclass correlation coefficient (ICC), which ranges from 0 to 1. ICC values are interpreted as follows: below 0.5 indicates poor reliability, 0.51–0.70 represents moderate reliability, 0.71–0.90 demonstrates good reliability, and above 0.90 presents excellent reliability [35].

#### Face validity

Face validity is a preliminary assessment of whether an instrument accurately reflects the intended concept [36]. The evaluation criteria for the instrument included grammar, language, spelling, sentence structure, writing style, format, and content. Respondents were asked to provide feedback by indicating their agreement or disagreement. An “agree” response suggested that the item was well-structured and aligned with the intended measurements. Additionally, respondents provided comments and suggestions for improvement [37].

#### Internal consistency

Internal consistency assesses the degree to which various components of an instrument uniformly measure the intended construct. This metric evaluates inter-item correlations within a test, reflecting their cohesiveness in capturing the target concept. To assess internal consistency, Cronbach’s alpha coefficient was employed. Following the guidelines [38], a coefficient between 0.60 and 0.69 was deemed acceptable, between 0.70 and 0.89 was considered good, and between 0.90 and 1.0 was regarded as excellent.

#### Criterion validity

To assess criterion validity, an examination was conducted on the relationship between the subjective measurement of halitosis and its impact on quality of life. Self-reported halitosis served as a subjective measure, reflecting individuals’ perceptions of their oral malodor. Pearson’s correlation coefficient was employed to evaluate the validity of the T-HALT scores by analyzing their association with scores indicating the level of self-reported halitosis.

#### Discriminant validity

To assess discriminant validity, an analysis was conducted between the objective measurement of halitosis and its impact on quality of life. The relationship between objective halitosis measurements (VSC levels from OralChroma™) and quality of life was examined. Based on previous evidence, VSC levels did not significantly impact quality of life, indicating that even high VSC levels do not necessarily diminish quality of life, and low VSC levels can still significantly affect it [39]. In other words, halitosis-related quality of life should be measured independently of objective halitosis severity. Pearson's correlation was performed to confirm the discriminant validity of T-HALT, showing it measured quality of life independently of VSC levels [40].

#### Construct validity

Construct validity was assessed using exploratory factor analysis (EFA) and subsequently confirmatory factor analysis (CFA) on data obtained from the HALT section of the questionnaire. Factor analysis was employed to reduce data dimensionality and extract principal components that account for the majority of variance in factors related to the impact of bad breath on quality of life. Before proceeding with EFA, key assumptions were verified to confirm the suitability of the correlation matrix. The randomness of the correlation matrix was assessed using Bartlett’s test of sphericity. Furthermore, the Kaiser-Meyer-Olkin (KMO) was employed to evaluate sampling adequacy, with a requirement that all variables attain a KMO value greater than 0.5. Given the expected correlation between questionnaire items, the oblique rotation method (Promax with Kaiser Normalization) was utilized to rotate factors. Eigenvalues were used to select principal components, with each selected component required to have an Eigenvalue greater than 1. Data extraction was conducted using the Principal Component Analysis (PCA) method, which is particularly suitable for recovering relatively weak factors when the assumptions of normality are violated. Subsequently, CFA was employed to validate the factorial validity of the models derived from the EFA results [41]. The model fit was assessed using absolute fit indices including Chi-Square (x^2^), where a well-fitting model should have a non-significant (x^2^ > 0.05) and a small Chi-square to degree of freedom ratio (CMIN /df < 2.0). Other absolute fit indices used were Goodness of Fit Index (GFI ≥ 0.90), Adjusted Goodness of Fit Index (AGFI ≥ 0.80), Root Mean Square Error of Approximation (RMSEA < 0.08), and Root Mean Square Residual (RMR < 0.08). Additionally, incremental fit indices were estimated, including Incremental Fit Index (IFI ≥ 0.90), Comparative Fit Index (CFI ≥ 0.90), Normed Fit Index (NFI ≥ 0.90), and Tucker Lewis Index (TLI ≥ 0.90) [42, 43]. Items in the questionnaire were removed by using CFA to evaluate the hypothesized factor structure or model by checking its fit with the data, and it was repeated until a satisfactory model fit was obtained.

### Ethical considerations

This research protocol was approved by the Faculty of Dentistry/Faculty of Pharmacy, Mahidol University, Institutional Review Board (COA.No.MU-DT/PY-IRB 2023/036.1506). Informed consent was obtained from all participants prior to data collection.

## Results

### Research participants

The study cohort consisted of 200 individuals with a mean age of 48.55 years (range: 18–88 years) and a mean BMI of 23.74 kg/m². Most participants were female, which seemed to reflect the actual proportion of the population. Furthermore, over half of the participants indicated having medical conditions requiring medication. The majority of participants were non-smokers and non-drinkers. Approximately 70% reported twice-daily tooth brushing and daily tongue brushing, and attending annual dental check-up. Nearly 80% utilized oral hygiene aids, particularly dental floss. Dental prosthesis wear was reported by 40% of participants, while 30% used orthodontic appliances Demographic data of research participants are demonstrated in Table 1.

**Table 1:** Demographics of research participants

Variables		*n*	%
**Age**	18–40 (Early adulthood)	72	36
	41–60 (Middle adulthood	62	31
	> 61 (Late adulthood)	66	33
**Sex**	Male	70	35
	Female	130	65
**BMI**	< 18.5 (Underweight)	16	8
	18.5–24.9 (Normal)	109	54.5
	25-29.9 (Overweight)	62	31
	>30 (Obese)	13	6.5
**Medical conditions**	No	109	54.5
	Yes	91	45.5
**Medication**	No	113	56.5
	Yes	87	43.5
**Occupation**	Unemployed	76	38
	Education and service professionals	37	18.5
	Healthcare professionals	7	3.5
	Business and sales professionals	22	11
	Administrative and office workers	54	27
	Other	4	2
**Level of education**	Undergraduate Degree	41	20.5
	Bachelor’s degree	124	62
	Postgraduate Degree	35	17.5
**Alcohol consumption**	No alcohol consumption	156	78
	1–5 days/month	33	16.5
	6–19 days/month	4	2
	More than 20 days/month	7	0.5
**Smoking**	No smoking	195	97.5
	Fewer than 10 cigarettes/day	4	2
	More than 10 cigarettes/day	1	0.5
**Annual dental visits**	Yes	147	73.5
	No	53	26.5
**Tooth brushing frequency**	Not regularly	0	0
	Once per day	9	4.5
	Twice per day	154	77
	Three times per day	30	15
	> Three times per day	7	3.5
**Tongue brushing**	Yes	147	73.5
	No	53	26.5
**Oral hygiene aids used**	None	36	18
	Dental floss	103	51.5
	Interproximal brush	45	22.5
	Gauze	1	0.5
	Toothpick	45	22.5
	Mouthwash	64	32
	Tongue scraper	2	1
**Wearing of dental prosthesis**	Yes	84	42
	No	116	58
**Wearing of orthodontic appliance**	Yes	57	28.5
	No	143	71.5

### Halitosis and halitosis-related quality of life

The OralChroma™ objectively evaluated halitosis, measuring VSCs at a mean of 129.63 ± 125.10 parts per billion. Participants also evaluated self-perceived halitosis using a 6-point Likert scale, with a mean score of 2.25 ± 1.07. Additionally, the T-HALT evaluation yielded a mean score of 16.37 ± 15.10 (Table 2).

**Table 2:** Objective and subjective measurements of halitosis and halitosis-related quality of life

Measurement	Mean (SD)
Objective measurement of halitosis (OralChroma™)	129.63 (125.10)
Subjective measurement of halitosis (Self-perceived halitosis)	2.25 (1.07)
Halitosis impacts quality of life (T-HALT)	16.37 (15.10)

### Reliability tests

#### Test-retest reliability

Test–retest reliability was evaluated for a subset of 30 patients, with retesting conducted after a 1-week interval (Table 3). The ICC for the overall T-HALT was 0.886 (95% CI = 0.672–0.784), while that of overall self-perceived halitosis was 0.783 (95% CI = 0.482–0.789). Two items were subsequently deleted due to low ICC values. As a result, the adjusted version of self-perceived halitosis included only four items. The ICC for the revised self-perceived halitosis was 0.824. For further details on each item, see Supplementary 5.

**Table 3:** Internal consistency and test-retest reliability

Variable	Phase 1		Phase 2
	ICC	Coefficient Alpha	Coefficient Alpha
**Self-perceived halitosis**	0.783	0.846	N/A
**Self-perceived halitosis (Adjusted)**	0.824	0.846	0.849
**T-HALT**	0.886	0.940	0.943

#### Internal consistency

The Cronbach’s alphas for T-HALT were 0.940 in Phase 1 and 0.943 in Phase 2, indicating excellent internal consistency, while those of self-perceived halitosis were 0.846 in Phase 1 and 0.849 in Phase 2 (Table 3). The Cronbach’s alpha values, if an item were deleted, ranged from 0.933 to 0.945 in Phase 1 and from 0.937 to 0.947 in Phase 2. The mean item scores from Phase 1 ranged from a low of 0.23 ± 0.63 (Item 10) to a high of 1.87 ± 1.33 (Item 4), while those from Phase 2 ranged from a low of 0.31 ± 0.79 (Item 19) to a high of 1.91 ± 1.36 (Item 4). The corrected item–total correlation was generally moderate to strong across the items, with values ranging from 0.31 (Item 1) to 0.82 (Items 14, Item 16) in Phase 2, and from 0.22 (Item 7) to 0.82 (Item 9, Item 13) in Phase 1. Please refer to Supplementary 5 for details on Phase 1 and Supplementary 6 for details on Phase 2.

### Validity tests

#### Criterion and discriminant validity

The correlations between self-perceived halitosis and the VSCs from OralChroma™ on T-HALT results were examined using Pearson’s correlation to determine criterion and discriminant validity, respectively. The results of criterion validity demonstrated a significant relationship of self-perceived halitosis on T-HALT both in Phase 1 (0.414, *P* < 0.05) and Phase 2 (0.503, *P* < 001). However, the summation of VSCs from OralChroma™ did not have a significant correlation with T-HALT (Table 4).

**Table 4:** Criterion and discrimination validity

Variables	Phase 1		Phase 2	
	Coefficient	*P*-value	Coefficient	*P*-value
**Self-perceived halitosis**	0.414	0.023*	0.503	< 0.001***
**OralChroma™**	0.144	0.447	0.071	0.320

* The significance level was taken at *P *< 0.05

*** The significance level was taken at *P *< 0.001

#### Construct validity

##### Exploratory factor analysis

Analysis of sampling adequacy using the KMO measure yielded a value of 0.934, indicating excellent factorability of the correlation matrix. The Bartlett’s Test of Sphericity produced a chi-square statistic of 2971.465 (df = 190, *P* < 0.001), confirming that the variables were sufficiently interrelated for factor analysis of the T-HALT instrument. EFA was conducted using Promax rotation with Kaiser normalization (Table 5). The rotated component matrix identified a structure comprising four factors, with eigenvalues spanning from 1.046 to 10.235. Factor loadings revealed a range of 0.647 to 0.887, all exceeding the 0.60 suggested threshold [44]. The EFA identified four distinct factors in the T-HALT instrument: (1) Emotional limitations (Items 4, 5, 10, 11, 12, and 20); (2) Personal and social disabilities (Items 8, 14, 15, 16, 17, and 19); (3) Functional limitations (Items 6, 9, 13, and 18); and Physical limitations (Items 1, 2, 3, and 7). These factors accounted for 51.17%, 7.69%, 6.13%, and 5.23% of the total variance, respectively, explaining a cumulative 70.22% of the variance in the data.

**Table 5:** Factor loading of T-HALT

Items	Factor loading			
	Emotional limitations	Personal and social disabilities	Functional limitations	Physical limitations
**Item 1**				0.798
**Item 2**				0.647
**Item 3**				0.681
**Item 4**	0.755			
**Item 5**	0.809			
**Item 6**			0.712	
**Item 7**				0.728
**Item 8**		0.767		
**Item 9**			0.858	
**Item 10**	0.767			
**Item 11**	0.847			
**Item 12**	0.813			
**Item 13**			0.820	
**Item 14**		0.868		
**Item 15**		0.865		
**Item 16**		0.830		
**Item 17**		0.728		
**Item 18**			0.874	
**Item 19**		0.887		
**Item 20**	0.734			
**Eigenvalues**	10.235	1.538	1.226	1.046
**% of Variance**	51.17%	7.69%	6.13%	5.23%
**Cumulative %**	51.17%	58.86%	64.99%	70.22%

##### Confirmatory factor analysis

***First order confirmatory factor analysis***

CFA was conducted to assess the construct validity of the four factors identified in the EFA. The analysis revealed that standardized factor loadings varied across the models: (1) Emotional limitations: 0.58 to 0.82; (2) Personal and social disabilities: 0.64 to 0.92; (3) Functional limitations: 0.58 to 0.86; and (4) Physical limitations: 0.30 to 0.70. For the first three factors, all model fit indices met the requisite criteria (Table 6), providing robust evidence for their construct validity. Detailed factor loadings are presented in Supplementary 7. However, the Physical limitations factor demonstrated some psychometric weaknesses. Several of its standardized factor loadings fell below the acceptable threshold of 0.50 [45]. Additionally, both the Average Variance Extracted (AVE) and Composite Reliability (CR) values for this factor were below acceptable criteria.

**Table 6:** Model fit indices for measurement models

Model Fit indices	Criteria	First order measurement model				Second order measurement model	
		'Emotional limitations' model	'Personal and social disabilities' model	'Functional limitations' model	'Physical limitations' model	Model before adjusting the modification index	Model after adjusting the modification index
**X** ^**2**^	-	6.128	8.085	0.263	1.134	212.229	80.352
**DF**	-	4	5	1	2	119.0	75
**P**	> 0.05	0.19	0.152	0.608	0.567	0.000	0.315
**CMIN/df**	< 2.0	1.532	1.617	0.263	0.567	1.783	1.071
**CFI**	≥ 0.90	0.997	0.996	1	1	0.968	0.998
**NFI**	≥ 0.90	0.992	0.990	0.999	0.979	0.931	0.973
**GFI**	≥ 0.90	0.990	0.987	0.999	0.997	0.898	0.958
**AGFI**	≥ 0.80	0.946	0.947	0.993	0.986	0.820	0.904
**IFI**	≥ 0.90	0.997	0.996	1.003	1.017	0.969	0.998
**RMSEA**	< 0.08	0.052	0.056	0.000	0.000	0.063	0.019
**RMR**	< 0.08	0.029	0.016	0.006	0.021	0.053	0.034
**TLI**	≥ 0.90	0.989	0.988	1.016	1.055	0.949	0.996
**AVE**	> 0.5	0.538	0.615	0.521	0.24	-	-
**CR**	> 0.6	0.873	0.904	0.810	0.532	-	-

*x*^*2*^ Chi-Square, *DF *Degrees of freedom, *CMIN/df *Chi-square to degree of freedom ratio, *CFI* Comparative fit index, *NFI* Normed fit index, *GFI *Goodness of fit index, *AGFI* Adjusted goodness of fit index, *IFI* Incremental fit index, *RMSEA* Root mean square error, *RMR* Root mean square residual, *TLI* Tucker-Lewis index, *AVE* Average variance extracted, *CR* Composite reliability

***Second order confirmatory factor analysis***

The second-order CFA identified four constructs comprising 20 items. Initial model fit indices are presented in Table 6. Two indices failed to meet acceptable thresholds: the Goodness of Fit Index (GFI = 0.898, threshold ≥ 0.90) and the Root Mean Square Residual (RMR = 0.053, threshold ≤ 0.08). Furthermore, standardized factor loadings for Items 1 (0.33) and 7 (0.40) were below the acceptable threshold of 0.50 [46]. Consequently, these two items were removed from the model (see Supplementary 8). The model was then refined using the modification index (MI) method, which involved adding covariances within construct indicators and adjusting relationship paths between constructs (see Supplementary 9). Post-adjustment model fit indices are presented in Table 6, demonstrating improved model fit. The final structure and psychometric properties of the adjusted model are summarized in Table 7.

**Table 7:** Analysis statistics of the second order confirmatory factor of the T-HALT model after adjusting the modification index

Variable		Factor loading	Error	t-value	*R* ^2^	AVE	CR	MSV
**Emotional limitations**	Item 4 (Parameters)	0.76	-	-	56.0%	0.619	0.907	0.987
	Item 5	0.75	0.07	14.990***	64.0%			
	Item 10	0.75	0.08	8.814***	72.0%			
	Item 11	0.80	0.08	9.074***	65.0%			
	Item 12	0.85	0.10	10.046***	58.0%			
	Item 20	0.81	0.09	9.936***	57.0%			
**Personal and social disabilities**	Item 8 (Parameters)	0.74	-	-	55.0%	0.595	0.897	0.933
	Item 14	0.85	0.11	12.239***	73.0%			
	Item 15	0.79	0.08	11.318***	63.0%			
	Item 16	0.83	0.10	11.821***	69.0%			
	Item 17	0.72	0.10	9.509***	52.0%			
	Item 19	0.67	0.07	9.446***	46.0%			
**Functional limitations**	Item 6	0.63	0.07	10.384***	40.0%	0.543	0.823	0.987
	Item 9 (Parameters)	0.88	-	-	77.0%			
	Item 13	0.71	0.07	12.107***	50.0%			
	Item 18	0.70	0.06	10.815***	50.0%			
**Physical limitations**	Item 2 (Parameters)	0.62	-	-	38.0%	0.362	0.531	0.519
	Item 3	0.59	0.16	5.279***	34.0%			

*** Statistical significance was taken at *P*< 0.001

## Discussion

The validity and reliability of OHRQoL questionnaires are fundamental to their efficacy and scientific value in oral health research. Validity (content, face, criterion, discriminant, and construct validity) ensures that an instrument accurately reflects the intended construct and its various dimensions [47], while reliability (internal consistency and test-retest reliability) pertains to the consistency of measurement across time and items [48, 49]. A valid and reliable OHRQoL instrument produces consistent results across different occasions and populations, provided the underlying condition remains stable [50]. In this context, our study on T-HALT addresses a critical need in the field by providing a rigorously validated and reliable instrument for assessing halitosis-related quality of life in Thai populations. This research not only contributes to the growing body of culturally adapted OHRQoL measures but also offers a valuable tool for clinicians and researchers studying the impact of halitosis on quality of life in Thailand.

T-HALT demonstrated excellent reliability across cultural adaptations. Internal consistency was high (Cronbach’s alphas = 0.940 in Phase 1 and 0.943 in Phase 2), comparable to the Cronbach’s alpha of 0.93 in the original HALT [12]. This indicates strong reliability across cultural adaptations. However, item analysis revealed that removing Items 1, 3, and 7 would slightly increase Cronbach’s alpha (Supplementary 6), potentially due to their reduced relevance in the Thai context. These items relate to less common external causes of halitosis, such as respiratory issues of the sinuses, tonsils, and nose [51], aligning with findings from the Chinese HALT validation [38]. The self-perceived halitosis subscale showed good internal consistency (Cronbach’s alpha = 0.849), with all items exceeding the recommended corrected item-total correlation threshold of 0.2 [52]. Test-retest reliability was excellent, with ICC values of 0.824 and 0.886 for self-perceived halitosis and T-HALT, respectively [35]. While these results support T-HALT’s reliability, future research could explore a shortened version excluding Items 1, 3, and 7 to enhance cultural specificity in Thai and other Asian populations.

Self-perceived halitosis significantly influenced quality of life, even without objective evidence from gas chromatography. This study distinguishes between measurable VSCs and subjective halitosis experience, as diagnosis or treatment might be unnecessary if neither the patient nor their social environment reports concerns, even with elevated VSC levels [53]. Consequently, gas chromatography was used in this study to assess discriminant validity and self-perceived halitosis for criterion validity. Our findings revealed a strong positive relationship between self-perceived halitosis and T-HALT scores. Interestingly, VSC measurements did not significantly correlate with T-HALT scores. T-HALT scores were associated with self-perceived halitosis, although they did not confirm its presence objectively. Prolonged exposure to an odor can lead to olfactory adaptation, potentially causing a discrepancy between subjective perceptions and objective measurements of halitosis [54, 55]. These associations demonstrate the tool’s efficacy in assessing individuals’ perceptions of breath odor and its impact on quality of life. This distinction underscores the importance of addressing psychological and social aspects of halitosis perception in patient care, highlighting the complex interplay between objective measures and subjective experiences in halitosis assessment.

The construct validity of T-HALT was assessed through factor analysis. EFA consistently identified four constructs, aligning with previous studies [15, 16], although the specific items associated with these constructs varied across different cultural adaptations. This variability underscores the importance of cultural context in halitosis-related quality of life measures and necessitated a rigorous validation process through CFA. The CFA approach in this study involved two sequential models. The initial model comprised four factors: Emotional limitations (Items 4, 5, 10, 11, 12, and 20), Personal and social disabilities (Items 8, 14, 15, 16, 17, and 19), Functional limitations (Items 6, 9, 13, and 18), and Physical limitations (Items 1, 2, 3, and 7). This structure broadly aligns with the conceptual framework of halitosis impact on quality of life [56]. However, the standardized factor loadings for Items 1 (0.33) and 7 (0.40) fell below the acceptable threshold of 0.50 [46], indicating poor fit with their designated construct. Consequently, these two items were excluded in the second model, potentially improving the instrument’s cultural specificity and psychometric properties.

T-HALT demonstrates effectiveness as a unidimensional questionnaire for Thai populations. However, its multidimensional application requires modifications, particularly the removal of Items 1 and 7, which showed poor psychometric properties. Further refinement is needed to improve the distribution of items across constructs, ensuring balanced representation of all dimensions. These findings highlight the importance of cultural adaptation to make the questionnaire appropriate and meaningful in the target culture [57]. The necessary modifications to T-HALT underscore the need for careful consideration of cultural nuances in translating and adapting health-related quality of life instruments. Future studies should focus on validating the modified T-HALT in diverse Thai populations, exploring the impact of removing Items 1 and 7 on the instrument’s overall validity and reliability, and investigating potential new items that might better capture the multidimensional nature of halitosis-related quality of life in the Thai context. These efforts would further enhance the effectiveness of T-HALT and ensure its applicability across various Thai demographics and regions.

A significant strength of this study is the rigorous validation and reliability processes, resulting in a robust questionnaire capable of providing actionable insights. One notable strength was the comprehensive approach to factor analysis, where EFA was initially conducted to identify the underlying factor structure and ensure alignment with existing literature, while CFA was employed to validate and confirm the factor structure identified by EFA. This sequential approach allowed for a thorough examination of the questionnaire’s factor structure, providing valuable insights into its validity. However, a limitation of this study is the use of the same sample for both EFA and CFA. Ideally, distinct samples are recommended for EFA and CFA to mitigate potential biases and ensure robust factor structure validation. Although using a single sample for both analyses provided a comprehensive preliminary and confirmatory assessment, it may introduce limitations in the robustness of the factor structure. Future research should consider using separate samples for EFA and CFA to enhance the validity and reliability of the findings.

Although the diverse participant pool recruited from the Dental Hospital, Faculty of Dentistry, Mahidol University, can enhance T-HALT’s applicability across various demographic groups, the single-site recruitment may limit generalizability. To address this limitation and further strengthen T-HALT, future research should include participants from various regions. Additionally, exploring the analysis of individual VSCs separately could provide deeper insights into specific contributors to halitosis and enhance T-HALT’s precision. The current study included participants who had taken antibiotics to maintain a representative sample of the general population. Future research should investigate the impact of antibiotics on halitosis, informing whether separate analyses or exclusion criteria are warranted for more accurate assessments. Such efforts would contribute significantly to the development of a culturally sensitive instrument for assessing halitosis-related quality of life in Thai populations and offer potential adaptations for other cultural contexts.

##  Conclusion

T-HALT has been successfully validated, demonstrating its reliability and validity for assessing halitosis-related quality of life in Thai populations. T-HALT shows excellent psychometric properties, including high internal consistency and test-retest reliability. While effective as a unidimensional questionnaire, its multidimensional application requires some modifications, particularly the removal of a few items. These findings highlight the need for cultural adaptation in patient-reported outcome measures. Future research should focus on validating the modified T-HALT across diverse Thai populations and exploring new items to enhance its cultural specificity, thereby contributing to improved assessment of halitosis-related quality of life in Thailand and other similar cultural contexts.

## Supplementary Information

Supplementary Material 1.

## Data Availability

The data that support the findings of this study are available from the corresponding author, up-on reasonable request. The data are not publicly available due to information that could compromise the privacy of research participants.
