# Supplementary material for: Development and validation of the Thai Halitosis Associated Life-Quality Test (T-HALT): an evaluation of psychometric properties
Source: BMC Oral Health. 2024 Oct 8;24:1196. doi: 10.1186/s12903-024-04926-y (PMC11462733; doi:10.1186/s12903-024-04926-y)

Supplementary 1: English version of the questionnaire

Part 1: General information

1. Age: ………………….............. years

2. Sex: ❑ Male ❑ Female

3. Weight: ……………………………….……......…kg

4. Height: ……………………………………....... cm

5. Medical conditions: ………………………………………………………………….

6. Medication: ………………………………………………………………………….

7. Occupation: ....……………………………..…………………………………

8. Level of education:

❑ Undergraduate Degree

❑ Primary School

❑ Junior High School

❑ Senior High School

❑ Diploma

❑ Bachelor's degrees

❑ Postgraduate

❑ Other (Please specify) ....

9. Alcohol consumption:

❑ 1-5 days/month

❑ 6-19 days/month

❑ More than 20 days/month

❑ No alcohol consumption

10. Smoking:

❑ Fewer than 10 cigarettes/day

❑ More than 10 cigarettes/day

❑ No smoking

11. Annual dental visits:

- ☐ Yes
- ☐ No

12. Tooth brushing frequency:

- ☐ Not regularly
- ☐ Once per day
- ☐ Twice per day
- ☐ Three times per day
- ☐ More than three times per day

13. Tongue brushing:

- ☐ Yes
- ☐ No

14. Oral hygiene aids used:

- ☐ None
- ☐ Dental floss
- ☐ Interproximal brush
- ☐ Gauze
- ☐ Toothpick
- ☐ Mouthwash
- ☐ Other (Please specify) ....

15. Wearing of dental prosthesis

- ☐ No
- ☐ Yes (Please specify) ....

16. Wearing of orthodontic appliance

- ☐ No
- ☐ Yes (Please specify) ....

Part 2: Self-perceived Halitosis

Please answer the questionnaire by circling the number that most closely matches your opinion

You realize that you have a problem with bad breath during the following periods		No problem	Very mild problem	Mild problem	Moderate problem	Severe problem	Extreme problem
1	Wake up	0	1	2	3	4	5
2	Day time	0	1	2	3	4	5
3	Thirsty	0	1	2	3	4	5
4	Wearing a mask	0	1	2	3	4	5

Please assess your bad breath by closing your mouth for 3 minutes, then cover your mouth and nose with your hands, exhale through your mouth and inhale through your nose. Then assess your bad breath by marking the straight line below.

0 ______________________________ 10

No malodor — Strong malodor

Part 3: Halitosis-associated quality of life

This part is adopted from the original version of the HALT questionnaire.

(Kizhner, V.; Xu, D.; Krespi, Y.P. A new tool measuring oral malodor quality of life. Eur Arch Otorhinolaryngol 2011, 268, 1227-1232)

Below you will find a list of symptoms and social/ emotional consequences of your oral malodor. We would like to know more about these problems and would appreciate your answering the following questions to the best of your ability. There are no wrong or right answers, and only you can provide us with this information. Age………………… Gender M / F

	Consider how severe is the problem when you experience it and how frequently it happens, please rate each item how "bad" it is by circling the number that corresponds with how you feel using this scale.	No problem	Very mild problem	Mild or slight problem	Moderate problem	Severe problem	Problem as bad as it .
1	Mainly mouth breathing	0	1	2	3	4	5
2	Frequent tonsillar infections	0	1	2	3	4	5
3	Frequent sinus infections	0	1	2	3	4	5
4	Worrying about or self conscious about your mouth breath	0	1	2	3	4	5
5	Miserable or tense due to halitosis	0	1	2	3	4	5
6	Difficulty chewing or limiting certain food due to halitosis	0	1	2	3	4	5
7	Change of taste	0	1	2	3	4	5
8	Problems speaking (or mouth covering) due to halitosis	0	1	2	3	4	5
9	Appearance affected due to halitosis	0	1	2	3	4	5
10	Depressed due to mouth breath	0	1	2	3	4	5
11	Problems concentrating due to halitosis	0	1	2	3	4	5
12	Embarrassed due to halitosis	0	1	2	3	4	5
13	Spending time related to halitosis? (such as additional gum chewing, extra brushing etc.)	0	1	2	3	4	5
14	Talking from afar due to halitosis	0	1	2	3	4	5
15	Avoid going out due to halitosis	0	1	2	3	4	5
16	Communication problems due to halitosis	0	1	2	3	4	5
17	Mentioned about halitosis	0	1	2	3	4	5
18	Suffer financial loss due to halitosis	0	1	2	3	4	5
19	Suffer social/ personal loss due to halitosis	0	1	2	3	4	5
20	Reduced life satisfaction due to halitosis	0	1	2	3	4	5

Supplementary 2: Thai version of the questionnaire

ส่วนที่ 1: ข้อมูลทั่วไป

1. อายุ : .... ปี

2. เพศ: ❑ ชาย ❑ หญิง

3. น้ำหนัก : ....กิโลกรัม

4. ส่วนสูง : .... เซนติเมตร

5. โรคประจำตัว : ....

6. ยาที่รับประทาน : ....

7. อาชีพ : ....

8. ระดับการศึกษา :

❑ ต่ำกว่าประถมศึกษา

❑ ระดับประถมศึกษา

❑ ระดับมัธยมศึกษาตอนต้น

❑ ระดับมัธยมศึกษาตอนปลาย

❑ อนุปริญญา/ ปวส./ปวช./ปวท.

❑ ปริญญาตรี

❑ สูงกว่าปริญญาตรี

❑ อื่นๆ (โปรดระบุ).......................

9. การบริโภคเครื่องดื่มแอลกอฮอล์ :

❑ 1-5 วัน/เดือน

❑ 6-19 วัน/เดือน

❑ มากกว่า 20 วัน/เดือน

❑ ไม่ดื่ม

10. สูบบุหรี่ :

❑ สูบน้อยกว่า 10 มวน/วัน

❑ สูบตั้งแต่ 10 มวน/วัน ขึ้นไป

❑ ไม่สูบ

11. ท่านได้พบทันตแพทย์เป็นประจำทุกปี

❑ ใช่

❑ ไม่ใช่

12. ความถี่ในการแปรงฟัน

❑ ไม่ได้แปรงฟันทุกวัน

❑ 1 ครั้ง/วัน

❑ 2 ครั้ง/วัน

❑ 3 ครั้ง/วัน

❑ มากกว่า 3 ครั้ง/วัน

13. ท่านได้แปรงลิ้นหรือไม่

❑ แปรง

❑ ไม่แปรง

14. ท่านใช้อุปกรณ์เสริมในการทำความสะอาดช่องปาก

❑ ไม่ใช้อุปกรณ์เสริม

❑ ไหมขัดฟัน

❑ แปรงซอกฟัน

❑ ผ้าก๊อซ

❑ ไม้จิ้มฟัน

❑ น้ำยาบ้วนปาก

❑ อื่นๆ ....

15. ท่านใส่ฟันเทียมอยู่หรือไม่

❑ ไม่ใส่

❑ ใส่ ได้แก่...

16. ท่านใส่เครื่องมือจัดฟันอยู่หรือไม่

❑ ไม่ใส่

❑ ใส่ ได้แก่..

ส่วนที่ 2: แบบประเมินกลิ่นปากด้วยตนเอง

กรุณาตอบแบบสอบถามโดย วงกลมรอบตัวเลข ที่สอดคล้องกับความรู้สึกของท่านในช่องที่ตรงกับความเห็นของท่านมากที่สุด

	ท่านตระหนักว่าท่านมีปัญหากลิ่นปากในช่วงเวลาดังต่อไปนี้	ไม่มีปัญหา	มีปัญหาน้อยมาก	มีปัญหาน้อย	มีปัญหาปานกลาง	มีปัญหามาก	มีปัญหามากที่สุด
1	หลังตื่นนอน	0	1	2	3	4	5
2	ช่วงเวลากลางวัน	0	1	2	3	4	5
3	ตอนกระหายน้ำ	0	1	2	3	4	5
4	ตอนใส่หน้ากากอนามัย	0	1	2	3	4	5

กรุณาประเมินกลิ่นปากของท่านโดยปิดปากเป็นระยะเวลา 3 นาที จากนั้นใช้มือป้องปากและจมูก โดยให้มือแนบสนิทกับใบหน้า หายใจออกทางปากและหายใจเข้าทางจมูก แล้วประเมินกลิ่นปากของท่านโดยการทำเครื่องหมายตัดผ่านลงบนเส้นตรงด้านล่าง

0 ______________________________ 10

ไม่มี — มีกลิ่นเหม็นรุนแรง

ส่วนที่ 3: ความสัมพันธ์ของกลิ่นปากต่อคุณภาพชีวิต

แบบสอบถามดังต่อไปนี้แสดงลักษณะอาการ ผลกระทบทางสังคมและอารมณ์ที่เป็นผลจากการมีกลิ่นปากของท่านในช่วง 1 เดือนที่ผ่านมา ผู้วิจัยมีความต้องการทราบเพิ่มเติมเกี่ยวกับปัญหาดังกล่าว ขอให้ท่านตอบแบบสอบถามนี้ให้ดีที่สุดเท่าที่ท่านจะทำได้ โดยให้ตรงกับความคิดเห็นมากที่สุดเพียงข้อเดียว คำตอบนี้ไม่มีผิดหรือถูก และมีเพียงท่านเท่านั้นที่สามารถให้ข้อมูลนี้ได้

	ขอให้ท่านพิจารณาถึงระดับความรุนแรงและความถี่ของปัญหาที่ตรงกับปัญหากลิ่นปากของท่านมากที่สุด โดยการวงกลมรอบตัวเลขที่สอดคล้องกับความรู้สึกของท่าน	ไม่มีปัญหา	มีปัญหาน้อยมาก	มีปัญหาน้อย	มีปัญหาปานกลาง	มีปัญหามาก	มีปัญหามากที่สุด
1	ท่านหายใจทางปากเป็นส่วนใหญ่	0	1	2	3	4	5
2	ท่านมีภาวะต่อมทอนซิลอักเสบบ่อยครั้ง	0	1	2	3	4	5
3	ท่านมีภาวะไซนัสอักเสบบ่อยครั้ง	0	1	2	3	4	5
4	ท่านมีความกังวลเรื่องกลิ่นปากของตัวเอง หรือรู้สึกว่าตัวเองมีกลิ่นปาก	0	1	2	3	4	5
5	ท่านรู้สึกทุกข์ใจหรือเครียดจากปัญหากลิ่นปาก	0	1	2	3	4	5
6	ท่านเคี้ยวลำบากหรือจำกัดการบริโภคอาหารบางชนิดเนื่องจากปัญหากลิ่นปาก	0	1	2	3	4	5
7	ท่านมีการรับรสเปลี่ยนไป	0	1	2	3	4	5
8	ท่านมีปัญหาในการพูด (หรือต้องป้องปาก) เนื่องจากปัญหากลิ่นปาก	0	1	2	3	4	5
9	ภาพลักษณ์ของท่านได้รับผลกระทบจากปัญหากลิ่นปาก	0	1	2	3	4	5
10	ท่านรู้สึกซึมเศร้าจากกลิ่นปาก	0	1	2	3	4	5
11	ท่านมีปัญหาเรื่องสมาธิเนื่องจากปัญหากลิ่นปาก	0	1	2	3	4	5
12	ท่านรู้สึกอับอายเนื่องจากปัญหากลิ่นปาก	0	1	2	3	4	5
13	ท่านใช้เวลาไปกับปัญหากลิ่นปาก เช่น มีการเคี้ยวหมากฝรั่งมากขึ้น แปรงฟันมากขึ้น และอื่น ๆ	0	1	2	3	4	5
14	ท่านเว้นระยะห่างขณะสนทนาเนื่องจากปัญหากลิ่นปาก	0	1	2	3	4	5
15	ท่านหลีกเลี่ยงการออกไปข้างนอกเนื่องจากปัญหากลิ่นปาก	0	1	2	3	4	5
16	ท่านมีปัญหาในการสื่อสารเนื่องจากปัญหากลิ่นปาก	0	1	2	3	4	5
17	ท่านเคยถูกทักเรื่องปัญหากลิ่นปาก	0	1	2	3	4	5
18	ท่านประสบปัญหาด้านค่าใช้จ่ายเนื่องจากปัญหากลิ่นปาก	0	1	2	3	4	5
19	ท่านสูญเสียสังคมหรือความสัมพันธ์กับคนใกล้ชิดเนื่องจากปัญหากลิ่นปาก	0	1	2	3	4	5
20	ท่านมีความพึงพอใจในชีวิตลดลงเนื่องจากปัญหากลิ่นปาก	0	1	2	3	4	5

Supplementary 3: Original questionnaire with the final Thai version

Items	English version of HALT questionnaire	Thai version of HALT questionnaire
**Item 1**	Mainly mouth breathing	ท่านหายใจทางปากเป็นส่วนใหญ่
**Item 2**	Frequent tonsillar infections	ท่านมีภาวะต่อมทอนซิลอักเสบบ่อยครั้ง
**Item 3**	Frequent sinus infections	ท่านมีภาวะไซนัสอักเสบบ่อยครั้ง
**Item 4**	Worrying about or self conscious about your mouth breath	ท่านมีความกังวลเรื่องกลิ่นปากของตัวเอง หรือรู้สึกว่าตัวเองมีกลิ่นปาก
**Item 5**	Miserable or tense due to halitosis	ท่านรู้สึกทุกข์ใจหรือเครียดจากปัญหากลิ่นปาก
**Item 6**	Difficulty chewing or limiting certain food due to halitosis	ท่านเคี้ยวลำบากหรือจำกัดการบริโภคอาหารบางชนิดเนื่องจากปัญหากลิ่นปาก
**Item 7**	Change of taste	ท่านมีการรับรสเปลี่ยนไป
**Item 8**	Problems speaking (or mouth covering) due to halitosis	ท่านมีปัญหาในการพูด (หรือต้องป้องปาก) เนื่องจากปัญหากลิ่นปาก
**Item 9**	Appearance affected due to halitosis	ภาพลักษณ์ของท่านได้รับผลกระทบจากปัญหากลิ่นปาก
**Item 10**	Depressed due to mouth breath	ท่านรู้สึกซึมเศร้าจากกลิ่นปาก
**Item 11**	Problems concentrating due to halitosis	ท่านมีปัญหาเรื่องสมาธิเนื่องจากปัญหากลิ่นปาก
**Item 12**	Embarrassed due to halitosis	ท่านรู้สึกอับอายเนื่องจากปัญหากลิ่นปาก
**Item 13**	Spending time related to halitosis? (such as additional gum chewing, extra brushing etc.)	ท่านใช้เวลาไปกับปัญหากลิ่นปาก เช่น มีการเคี้ยวหมากฝรั่งมากขึ้น แปรงฟันมากขึ้น และอื่น ๆ
**Item 14**	Talking from afar due to halitosis	ท่านเว้นระยะห่างขณะสนทนาเนื่องจากปัญหากลิ่นปาก
**Item 15**	Avoid going out due to halitosis	ท่านหลีกเลี่ยงการออกไปข้างนอกเนื่องจากปัญหากลิ่นปาก
**Item 16**	Communication problems due to halitosis	ท่านมีปัญหาในการสื่อสารเนื่องจากปัญหากลิ่นปาก
**Item 17**	Mentioned about halitosis	ท่านเคยถูกทักเรื่องปัญหากลิ่นปาก
**Item 18**	Suffer financial loss due to halitosis	ท่านประสบปัญหาด้านค่าใช้จ่ายเนื่องจากปัญหากลิ่นปาก
**Item 19**	Suffer social/ personal loss due to halitosis	ท่านสูญเสียสังคมหรือความสัมพันธ์กับคนใกล้ชิดเนื่องจาก ปัญหากลิ่นปาก
**Item 20**	Reduced life satisfaction due to halitosis	ท่านมีความพึงพอใจในชีวิตลดลงเนื่องจากปัญหากลิ่นปาก

Supplementary 4: The results of content validity

	items	Expert 1	Expert 2	Expert 3	IOC
Self-perceived halitosis	1	+1	+1	+1	1.00
	2	+1	+1	+1	1.00
	3	+1	+1	+1	1.00
	4	+1	+1	+1	1.00
	5	+1	+1	+1	1.00
	6	+1	0	+1	0.66
T-HALT	1	+1	0	+1	0.66
	2	0	+1	+1	0.66
	3	+1	0	+1	0.66
	4	+1	0	+1	0.66
	5	+1	+1	+1	1.00
	6	+1	+1	+1	1.00
	7	0	+1	+1	0.66
	8	+1	+1	+1	1.00
	9	+1	+1	+1	1.00
	10	+1	+1	+1	1.00
	11	+1	+1	+1	1.00
	12	+1	+1	+1	1.00
	13	+1	+1	+1	1.00
	14	+1	+1	+1	1.00
	15	+1	+1	+1	1.00
	16	+1	+1	+1	1.00
	17	+1	+1	+1	1.00
	18	+1	+1	+1	1.00
	19	+1	+1	+1	1.00
	20	+1	+1	+1	1.00

Supplementary 5: Test-retest reliability and internal consistency in Phase 1

Test-retest reliability of individual items in self-perceived halitosis assessment

Items	Self-perceived halitosis	ICC
Item 1	Wake up	0.854
Item 2	Hungry	0.579
Item 3	Thirsty	0.783
Item 4	All day	0.628
Item 5	Wearing a mask	0.643
Item 6	Others	0.330

Test-retest reliability of individual items in T-HALT section

Items	HALT	ICC
Item 1	Mainly mouth breathing	0.377
Item 2	Frequent tonsillar infections	0.861
Item 3	Frequent sinus infections	0.668
Item 4	Worrying about or self conscious about your mouth breath	0.525
Item 5	Miserable or tense due to halitosis	0.660
Item 6	Difficulty chewing or limiting certain food due to halitosis	0.855
Item 7	Change of taste	0.793
Item 8	Problems speaking (or mouth covering) due to halitosis	0.760
Item 9	Appearance affected due to halitosis	0.743
Item 10	Depressed due to mouth breath	0.679
Item 11	Problems concentrating due to halitosis	0.708
Item 12	Embarrassed due to halitosis	0.853
Item 13	Spending time related to halitosis? (such as additional gum chewing, extra brushing etc.)	0.746
Item 14	Talking from afar due to halitosis	0.855
Item 15	Avoid going out due to halitosis	0.694
Item 16	Communication problems due to halitosis	0.702
Item 17	Mentioned about halitosis	0.819
Item 18	Suffer financial loss due to halitosis	0.675
Item 19	Suffer social/ personal loss due to halitosis	0.767
Item 20	Reduced life satisfaction due to halitosis	0.826

Internal consistency of the self-perceived halitosis assessment (Cronbach's alpha = 0.846)

Items	Mean±SD	Corrected item-total correlation	Cronbach's alpha if item deleted
Item 1	2.47 ± 1.25	0.536	0.837
Item 2	1.40 ± 1.48	0.733	0.798
Item 3	1.90 ± 1.52	0.792	0.784
Item 4	1.70 ±1.26	0.789	0.788
Item 5	1.97 ± 1.217	0.664	0.813
Item 6	0.20 ± 0.76	0.225	0.875

Internal consistency of the T-HALT section (Cronbach's alpha = 0.940)

Items	Mean±SD	Corrected item-total correlation	Cronbach's alpha if item deleted
Item 1	1.00 ± 1.02	0.68	0.936
Item 2	0.33 ± 0.76	0.65	0.937
Item 3	0.33 ± 0.80	0.47	0.939
Item 4	1.87 ± 1.33	0.60	0.938
Item 5	1.33 ± 1.32	0.77	0.934
Item 6	1.03 ± 1.35	0.66	0.937
Item 7	0.47 ± 1.11	0.22	0.945
Item 8	0.67 ± 0.88	0.62	0.937
Item 9	0.63 ± 0.77	0.82	0.934
Item 10	0.23 ± 0.63	0.79	0.936
Item 11	0.40 ± 0.72	0.73	0.936
Item 12	0.57 ± 1.07	0.75	0.935
Item 13	1.00 ± 1.29	0.82	0.933
Item 14	1.07 ± 1.02	0.73	0.935
Item 15	0.23 ± 0.77	0.80	0.935
Item 16	0.57 ± 1.10	0.74	0.935
Item 17	0.73 ± 0.74	0.40	0.940
Item 18	0.57 ± 1.07	0.53	0.939
Item 19	0.23 ± 0.68	0.77	0.936
Item 20	0.30 ± 0.70	0.80	0.935

Supplementary 6: Internal consistency in Phase 2

Internal consistency of the self-perceived halitosis assessment (Cronbach's alpha = 0.849)

Items	Mean±SD	Corrected item-total correlation	Cronbach's alpha if item deleted
Item 1	2.75 ± 1.32	0.620	0.838
Item 2	1.89 ± 1.17	0.724	0.797
Item 3	2.08 ± 1.32	0.708	0.800
Item 4	2.29 ± 1.31	0.710	0.799

Internal consistency of the T-HALT section (Cronbach's alpha = 0.943)

Items	Mean±SD	Corrected item-total correlation	Cronbach's alpha if item deleted
Item 1	1.18 ± 1.25	0.31	0.947
Item 2	0.53 ± 1.00	0.46	0.943
Item 3	0.36 ± 0.88	0.38	0.944
Item 4	1.91 ± 1.36	0.66	0.940
Item 5	1.57 ± 1.34	0.71	0.939
Item 6	0.94 ± 1.19	0.65	0.940
Item 7	0.46 ± 0.10	0.37	0.944
Item 8	0.79 ± 1.05	0.73	0.939
Item 9	0.96 ± 1.19	0.80	0.937
Item 10	0.44 ± 0.91	0.71	0.940
Item 11	0.54 ± 0.90	0.75	0.939
Item 12	1.01 ± 1.20	0.76	0.938
Item 13	1.15 ± 1.27	0.69	0.940
Item 14	1.23 ± 1.27	0.82	0.937
Item 15	0.46 ± 0.84	0.79	0.939
Item 16	0.67 ± 1.06	0.82	0.937
Item 17	0.74 ± 0.10	0.69	0.940
Item 18	0.44 ± 0.91	0.72	0.939
Item 19	0.31 ± 0.79	0.66	0.941
Item 20	0.69 ± 1.09	0.76	0.938

Supplementary 7: First order confirmatory factor analysis models

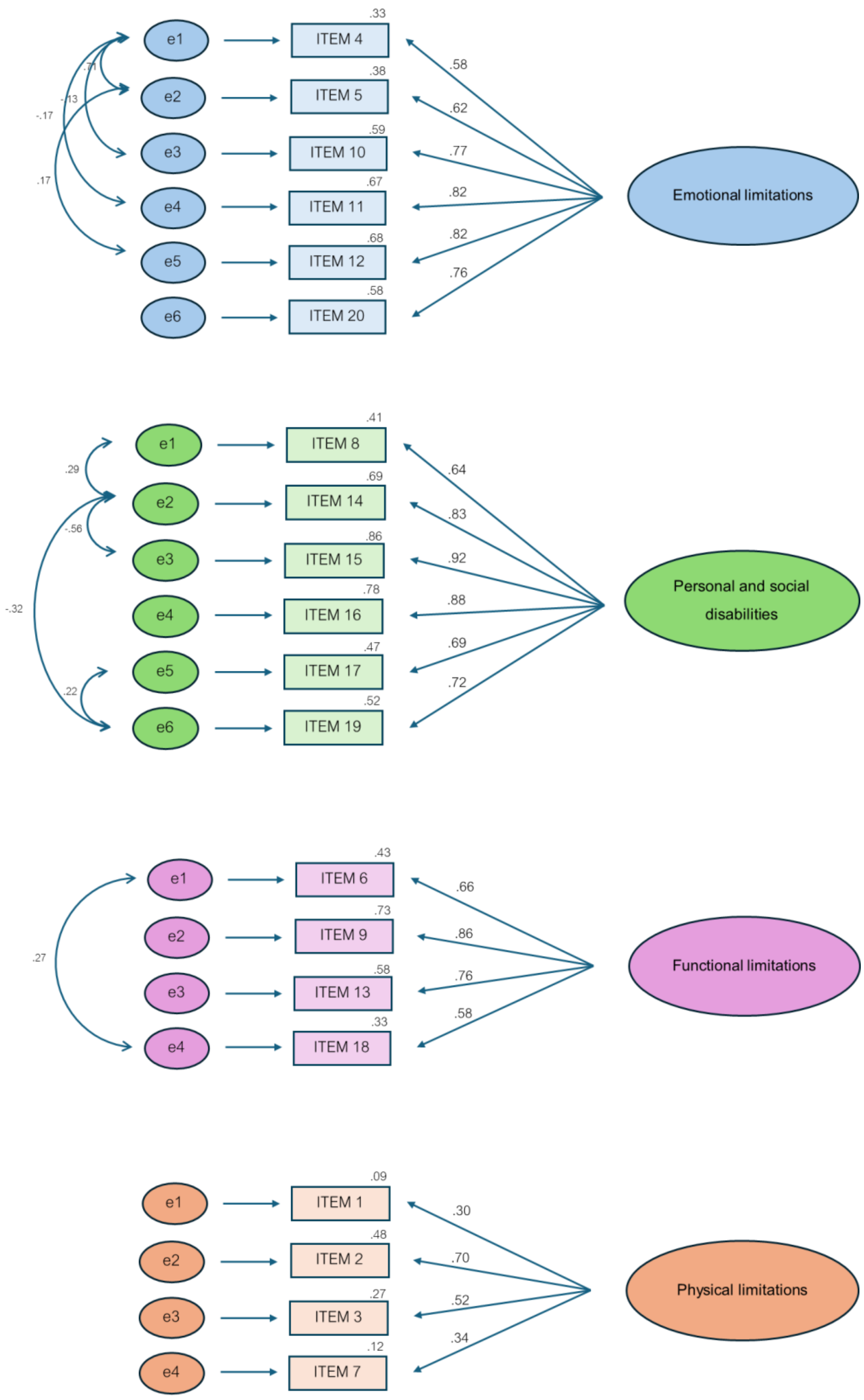

Supplementary 8: Second order confirmatory factor of the T-HALT model before adjusting the modification indices

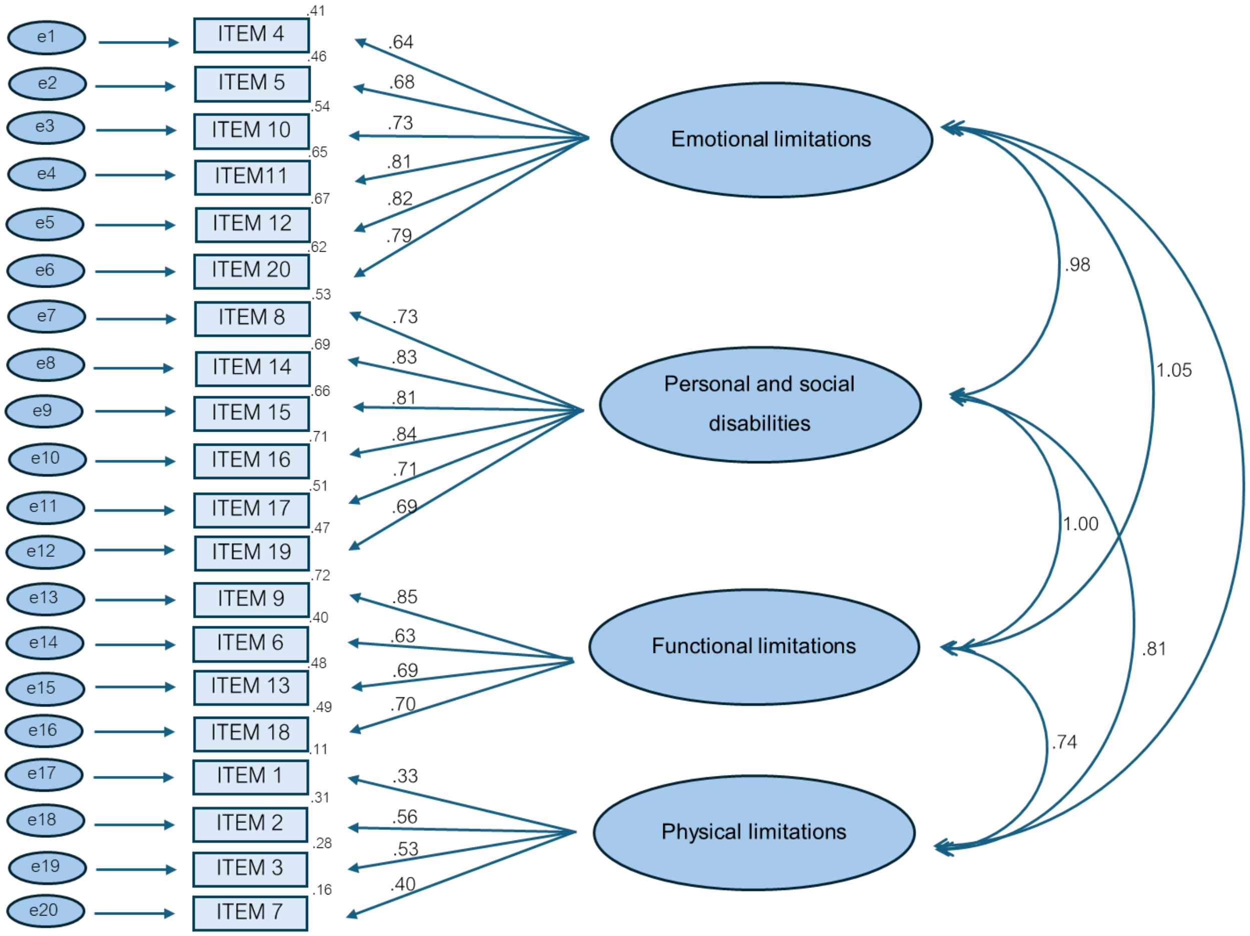

Supplementary 9: Second order confirmatory factor of the T-HALT model after adjusting the modification indices

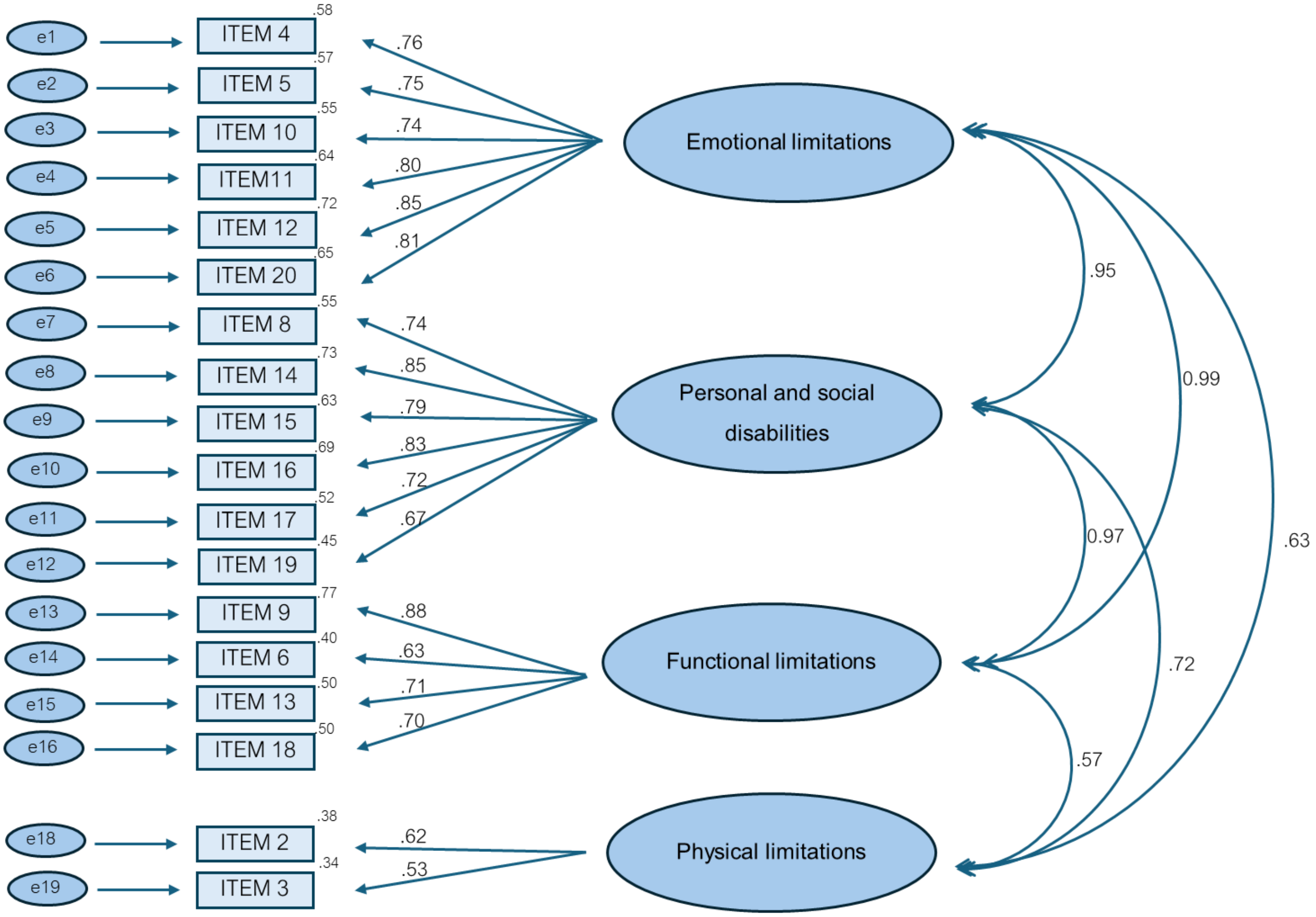

Supplement: Supplementary file 1 — Supplementary Material 1. [file 12903_2024_4926_MOESM1_ESM.pdf]
